# Evaluation N-Telopeptide (NTx) and calprotectin level in crevicular fluid with peri-implantitis

**DOI:** 10.6026/9732063002001183

**Published:** 2024-09-30

**Authors:** Ashwini Narayankar, Sagar Kumar Kashyap, Suman Yadav, Anas Abdul Khader, Anjaneya Dube, Pushkar Gupta, Nureldeen AN Elhammali, Abhaya Chandra Das

**Affiliations:** 1 Department of Prosthodontics, SB Patil Dental College, Bidar, Karnataka, India; 2 Intern, Kalinga Institute of Dental Sciences, Bhubaneswar, Odisha, India; 3 Department of Oral Surgery, ITS Dental College, Modinagar, UP, India; 4 Department of Periodontology and Implant Dentistry, College of Dentistry, Qassim University, Kingdom of Saudi Arabia; 5 Department of Oral and Maxillofacial Surgery, Hitkarini Dental College and Hospital, Jabalpur, MP, India; 6 Department of Prosthodontics and Crown & Bridge, Hitkarini Dental College and Hospital, Jabalpur, MP, India; 7 Department of Oral and Maxillofacial Surgery, Faculty of Dentistry, Sirte University, Libya; 8 Department of Periodontics and Oral Implantology, Institute of Dental Sciences, Siksha 'O' Anusandhan (Deemed to be University), Khandagiri Square, Bhubaneswar - 751030, Odisha, India

**Keywords:** Biomarkers, calprotectin, peri-implantitis

## Abstract

Following inflammation and bone loss near the implant site, peri-implantitis develops. N-Telopeptide (NTx) and Calprotectin are abundant in the crevicular fluids found in that area and are thought to be possible biomarkers. Therefore, it is of interest to evaluate the amounts of calprotectin and NTx in the peri-implant sulcular fluid (PISF) from implant sites with or without peri-implantitis. Twenty healthy individuals and twenty patients with peri-implantitis who had a single dental implant were included in the total of forty participants. For every patient, the peri-implant clinical parameters of gingival index (GI), probing depth (PD) and bleeding on probing (BOP) were ted. To evaluate the bone loss (BL), radiographic pictures of every implant were acquired. Using sterile paper strips, PISF was gathered in order to use the ELISA technique to measure the amounts of NTx and calprotectin. We examined the correlations between the levels of PISF, NTx and calprotectin with the peri-implant clinical indicators. In comparison to the healthy group, the peri-implantitis group had increased levels of NTx and calprotectin. In individuals with peri-implantitis, the levels of NTx and calprotectin in the PISF may be a promising indicator for bone loss and peri-implant inflammation.

## Background:

Dental implants are a popular method of replacing lost teeth. However, peri-implant illnesses continue to be a contributing factor in a growing percentage of implant failures in routine clinical dental practice. Peri-implant mucositis and peri-implantitis are the two types of peri-implant inflammation that have been recognised in the literature. According to the American Academy of Periodontology (AAP), suppuration and/or bleeding on probing (BOP) are clinical indicators of peri-implant mucositis. These symptoms are typically linked to probing depths (PDs) of ≥4 mm and shows evidence of radiographic bone loss beyond bone remodelling. A progressive, irreversible condition affecting the soft tissues and bone around osseointegrated dental implants is known as peri-implantitis [1-2]. Peri-implantitis is thought to impact 20% of patients and 10% of implants, according to previous studies [3]. For the diagnosis of peri-implantitis, a combination of clinical and radiographic markers, including pocket depth (PD), bleeding on probing (BOP), suppuration, mobility and marginal bone loss, are frequently used [4]. Biomarkers in peri-implant sulcular fluid (PISF) are being used to diagnose peri-implant disorders. When assessing inflammation, biomarkers are thought to be more accurate than clinical indications [5]. Biomarker to diagnose peri-implant inflammation is an invasive process. Leukocytes, macrophages and epithelial cells all produce the inflammation-related protein calprotectin, which is elevated in a number of inflammatory conditions, such as ulcerative colitis, rheumatoid arthritis and cystic fibrosis [6]. There has previous evidence of calprotectin (MRP 8/14) in gingival crevicular fluid (GCF). MMP-8 is a potent biomarker candidate for identifying alveolar bone degradation and is by far, the most thoroughly studied biomarker for periodontitis and peri-implantitis. Saliva or peri-implant crevicular fluid samples showed elevated MMP-8 levels, particularly in patients with concurrent periodontitis [7]. When Ata-Ali *et al.* compared the peri-implantitis locations to healthy peri-implant tissue; they found a markedly higher concentration of pro-inflammatory cytokines like IL-1β, IL-6, IL-10 and TNF-α. For peri-implant disorders, these proteins and components in GCF and PISF are thought to be diagnostic indicators [8]. Cross-linked N-telopeptide of type I collagen (NTx) is used frequent as a biomarker of bone resorption, to diagnose a number of disorders involving bone metabolism [9]. Very few studies are done on Calprotectin levels in PICF samples from healthy and peri-implant disease sites. Therefore, it is of interest to assess the calprotectin and N-Telopeptide levels in the peri-implant sulcular fluid (PISF) from implant sites with or without peri-implantitis.

## Materials and Methods:

The present clinical study was done in Department of Prosthodontics, SB Patil Dental College, Bidar. For the current clinical investigation, patients with single dental implants placed five to ten years ago with healthy implants and peri-implant disorders, were recruited. Total 40 patients (20 healthy and 20 with peri-implantitis with in age group of 25-55 years in both genders) were included for the study. The relevant authorities gave their ethical approval. Informed consent was obtained from all the participants. Individuals with both healthy and unhealthy dental implants who didn't had any antibiotic therapy within three months or no history of systemic inflammatory illnesses were eligible to participate in the study.

## Assessment of Peri-implant sulcular fluid (PISF):

Following the collection of PISF, probing depth (PD), bleeding on probing (BOP), bone loss and gingival index (GI) were evaluated as clinical markers. Using sterile paper strips, PISF samples were taken from the peri-implant sites that were in good health as well as those had peri-implantitis. Using the Periotron 8000 (Pro-Flow Inc., NY, USA), the volume of the gathered PISF was determined. Gingival index (GI) scores were assessed using modified versions of Löe and Silness's standard. Periodontal locations with PD ≥3 mm, BOP negative or positive and GI score ≥1 were classified as diseased sites with peri-implant disorders. The criteria for defining healthy implant sites were GI score of zero, BOP of zero and PD of less than 3 mm. Following collection, the fluid samples were transported to a lab for biochemical parameter testing. Using a ready-to-use solid-phase enzyme-linked immunosorbent assay (ELISA) (Life Techlogies (India) Pvt Ltd., India), the amount of calprotectin was determined. The instruction booklet states that a competitive ELISA (Ostex, osteomark, Seattle, WA, USA) was used to detect the NTx level. Calprotectin and NTx concentrations were given as nagrammes per micro-litre of PICF. The obtained data was statistically assessed using SPSS statistical software version 22.0, IBM, Chicago, IL, USA with Mann-Whitney U test and Fisher's exact test at P<0.05.

## Results:

Clinical findings of healthy and disease group were shown in Table 1. A significant increase in clinical findings for PD, BOP, Bone loss and GI scores observed for diseased over healthy group. N-Telopeptide (NTx) and Calprotectin levels in the peri-implantitis group were significantly higher than those in the healthy group (Table 2).

## Discussion:

Thanks to developments in the surgical techniques for dental implants, implant-based dental treatments are w commonly performed [10]. The current investigation showed that the peri-implantitis group's PICF sample quantities and concentrations of calprotectin and NTx were substantially greater than those of the healthy group. There was a positive correlation found between the mean levels of different periodontal markers evaluated clinically and the amounts of NTx and calprotectin inside the PICF. Our findings concur with those of earlier research conducted by Singh *et al.* and Kajiura *et al.* [2,11]. Calprotectin levels in PICF have the potential to serve as biomarkers for the detection of peri-implant disorders, according to the study's conclusion of Singh *et al.* [2]. Calprotectin and NTx levels in the PISF fluid from implant sites with or without peri-implantitis were evaluated by Alotaibi *et al.* They came to the conclusion that, in patients with peri-implantitis, the levels of NTx and calprotectin in the PISF may be a promising biomarker for peri-implant inflammation and bone loss [5]. The content of NTx and calprotectin in the gingival crevicular fluid (GCF) surrounding the implant sites was measured by Swarup *et al.* They came to the conclusion that both NTx and calprotectin might be employed as biomarkers [12]. Calprotectin and NTx in PICF have the potential to be biomarkers for the identification of peri-implant disorders, according to Sakamoto *et al.* study [10]. Our conclusions are supported with these studies. Increased bacterial activation of inflammatory cells and increased intracellular material release in periodontitis and gingivitis may be the cause of elevated calprotectin levels in diseased groups [13]. In PICF in peri-implities, Hentenaar *et al.* observed increased levels of IL-1β and MMP-8 in comparison to the healthy group [14]. According to Soysal *et al.* there was a substantial increase in IL-1β, IL-6, and sAA expression levels in peri-implantitis patients with high stress level evaluation scores [15]. A thorough evaluation by Delucchi *et al.* came to the conclusion that PICF sample might be a viable, repeatable, n-invasive type of liquid biopsy in implant dentistry [16]. A precise biomarker of bone resorption, NTx is formed when osteoclasts break down bone type I collagen due to cathepsin K. This product is quickly discharged into the bloodstream and urine [5]. Cumulative Interceptive Supportive Therapy (CIST) carefully selects the course of treatment or peri-implant diseases. Clinical periodontal factors are used in these criteria to diagnose peri-implant disorders [12]. GCF, an ultra-filtrate of plasma, offers benefits similar to a doctor collecting blood: it is invasive and site-specific for teeth. Significant bone loss may be predicted by NTx levels before it is by GCF and PCF calprotectin levels. Active periodontal deterioration is associated with elevated NTx levels [17]. The current study's limitations were a comparatively small sample size and the lack of an attempt to connect the severity of peri-implantitis with the levels of calprotectin and NTx. To validate the results, more research with larger samples is required.

## Conclusion:

Peri-implant illnesses were linked to PISF levels of calprotectin and N-telopeptide. NTx and calprotectin play a major role in the peri-implantitis diagnosis.

## Figures and Tables

**Table 1:** Clinical findings among groups

**Clinical findings**	**Healthy group**	**Peri implantitis group**	**p**
Probing depth - PD (mm)	2.08± 0.38	4.76± 1.23	
BOP-positive rate (%)	0.0± 0.0	35.0± 11.42	
Gingival index	0.0± 0.0	1.2± 0.42	0.001
Bone loss rate (%)	16.± 7.5	36.2± 15.12	

**Table 2:** Average calprotectin and N-Telopeptide levels and concentrations among both groups

**Biomarkers (Average)**	**Healthy group**	**Peri implantitis group**	p
Calprotectin (ng/site)	43.5± 0.27	174.5± 0.47	0.001
calprotectin concentration/µL of PICF	114.2± 0.54	231.2± 0.42	0.002
N-Telopeptide (NTx) (ng/site)	3.11± 0.32	6.58± 0.75	0.001
calprotectin concentration/µL of PICF	6.24± 0.43	9.65± 0.54	0.002
